# Meta-analysis of the Diagnostic Performance of Circulating MicroRNAs for Pancreatic Cancer

**DOI:** 10.7150/ijms.52706

**Published:** 2021-01-01

**Authors:** Cheng Peng, Jiale Wang, Wenzhe Gao, Lihua Huang, Yunfei Liu, Xia Li, Zhiqiang Li, Xiao Yu

**Affiliations:** 1 Department of Hepatopancreatobiliary Surgery, Third Xiangya Hospital, Central South University, Changsha 410013, Hunan, China.; 2 Center for Medical Experiments, Third Xiangya Hospital, Central South University, Changsha 410013, Hunan, China.; 3 Department of Endocrinology, Third Xiangya Hospital, Central South University, Changsha 410013, Hunan, China.

**Keywords:** pancreatic cancer, microRNAs, diagnosis, meta-analysis, circulating

## Abstract

**Background**: Numerous studies have suggested that differentially expressed miRNAs may be promising diagnostic markers for pancreatic cancer (PC), but the results are inconsistent. We aimed to summarize the diagnostic accuracy of circulating miRNAs, carbohydrate antigen 19-9 (CA19-9), and the combination of miRNAs and CA19-9.

**Material and Methods**: A literature search of online databases including PubMed, EMBASE, Cochrane Library, China National Knowledge Infrastructure (CNKI) and WanFang was conducted. Relative data were extracted from eligible included studies, and a meta-analysis was performed.

**Results**: A total of 46 studies involving 4,326 PC patients and 4,277 non-PC controls were included. The pooled sensitivity (SEN), specificity (SPE) and AUC of the circulating miRNAs for differentiating PC patients from non-PC controls were 0.79 (0.77-0.81), 0.77 (0.75-0.79), and 0.85 (0.81-0.87), respectively. The combination of miRNAs and CA19-9 greatly improved the SEN, SPE and AUC to 0.84 (0.80-0.87), 0.91 (0.89-0.93) and 0.94 (0.92-0.96), respectively. Moreover, circulating miRNAs also yielded an acceptable diagnostic accuracy for early-stage PC with a SEN of 0.79 (0.76-0.82), a SPE of 0.74 (0.68-0.79) and an AUC of 0.81 (0.77-0.84).

**Conclusion**: Circulating miRNAs exhibited satisfactory diagnostic performance for PC and even early-stage PC. The combination of circulating miRNAs and CA19-9 can further improve the diagnostic accuracy, providing a novel strategy for PC diagnosis.

## Background

Pancreatic cancer (PC) is a highly malignant digestive tract cancer characterized by strong invasiveness, a high recurrence rate and a poor prognosis. In 2018, there were approximately 458,918 new cases of PC worldwide, accounting for 2.5% of all new cases of cancer. Moreover, 432,242 patients died of PC, making it the seventh most common cause of cancer-related death^1^.

According to the recommendation of the US Preventive Services Task Force (USPSTF), screening for PC in asymptomatic individuals is currently not recommended^2^. However, early detection is valuable for individuals with risk factors (such as a familial history) for PC, as it can increase the resection rates and result in longer median survival^3^. Overall, the 5-year survival rate for PC is 9.3%, but it is largely determined by the stage at which PC is diagnosed. For PC patients with metastatic disease at the time of diagnosis, the 5-year survival rate is 2.9%. If regional disease is present, the 5-year survival rate is 12.4%. For patients with localized PC, the 5-year survival rate can increase to 37.4%^4^.

Conventional imaging methods, such as computed tomography (CT), magnetic resonance imaging (MRI), and ultrasound (US), and serum marker carbohydrate antigen 19-9 (CA19-9) have been widely used for the diagnosis of PC, but the diagnostic accuracy of these modalities may be suboptimal^5^, especially for early-stage PC 6. Endoscopic ultrasonography (EUS) has gradually come to be considered to be the most accurate diagnostic tool since it not only has higher sensitivity (SEN) and specificity (SPE) but can also facilitate the retrieval of specimens of suspected tissue by EUS-guided fine-needle aspiration (EUS-FNA) for pathological confirmation. However, its invasiveness and the risks attendant on sedation make it only suitable for selected individuals^7^, ^8^. In this clinical setting, liquid biopsy is of great interest from both scientific and clinical perspectives because of its noninvasiveness, higher sensitivity and cost-efficiency^9^. Numerous biomarkers derived from PC, including circulating tumor cells (CTCs), cell-free circulating tumor DNAs (cfDNAs), circulating microRNAs (miRNAs), long noncoding RNAs (lncRNAs), proteins and metabolites, circulating tumor extracellular vesicles (e.g., exosomes) and tumor-educated platelets (TEPs), can be detected by liquid biopsy^6^, ^9^.

MiRNAs are a class of noncoding RNAs 19-25 nucleotides in length that regulate protein synthesis at the posttranscriptional level and play an indispensable role in cancer initiation, proliferation, progression, metastasis and chemo-resistance^10^. Since 2010, many studies on the application of circulating miRNAs for the diagnosis of PC have been published. The purpose of the present study is to summarize these original studies and evaluate the diagnostic performance of circulating miRNAs for PC.

## Material and Methods

### Literature search and study selection

The process of the literature search and study selection was performed in accordance with the PRISMA guidelines^11^. A combination of MeSH terms and entry terms was used to search the mainstream databases, including PubMed, EMBASE and Cochrane Library. We also searched Chinese databases, including the China National Knowledge Infrastructure (CNKI) and WanFang databases. In addition, we conducted a manual search for potentially eligible studies based on the identified review articles' reference lists. The search terms we used included (1) circulating, circulatory, serum, plasma, blood; (2) microRNAs, miRNAs, miR, panel; (3) Pancreatic Neoplasms, Pancreatic Intraductal Neoplasms, pancreatic cancer, cancer of pancreas, pancreatic cancer, carcinoma, pancreas, pancreatic ductal adenocarcinoma or PDAC; and (4) screen, diagnosis, diagnostic, prediction, predict, monitor, detection, detect, predictor, marker, sensitivity, specificity, AUC. For example, our electronic search strategy for PubMed is detailed in the **Search strategy** Section at the end of this paper.

We obtained a substantial number of retrieved records through the database search and manual search. First, duplicated publications were removed by Endnote X9 software, and then we checked again to ensure that there were no duplicate records. The remaining articles were evaluated based on their titles and abstracts and were included for full-text assessment if they met all eligibility criteria based on the PICOS principle: (1) Participants: patients with PC; (2) Interventions: the detection of circulating miRNAs; (3) Comparisons: non-PC controls; (4) Outcomes: diagnostic SEN and specificity SPE, or the number of true positive (TP), false positive (FP), true negative (TN) and false negative (FN) results of the diagnostic test; and (5) Study design: diagnostic research. Any article was excluded during the full-text assessment if the data were found to be insufficient.

### Quality assessment

The quality of the included studies was assessed using the QUADAS-2 (Quality Assessment of Diagnostic Accuracy Studies 2) tool, which has been widely used since its publication in 2011^12^. The QUADAS-2 tool contains four domains, namely, "patient selection", "index test", "reference standard" and "flow and timing", which are used to objectively evaluate the risk of bias and concerns about the applicability of the included studies. The process of quality assessment and mapping was performed with RevMan 5.3 software.

### Data extraction and statistical analysis

The process of data extraction was independently completed by two researchers, with one extracting the data and another rechecking the data. The original data were extracted with a standardized form that included the following items: (1) general information about the article: the name of the first author, publication year, country; (2) research content: specimen type, conference test, the studied miRNAs or other markers and their corresponding expression levels in PC patients, normalization control; and (3) the data for the meta-analysis: the number of PC patients and non-PC controls, the composition of the control population, diagnostic SEN and SPE or the number of true positive (TP), false positive (FP), true negative (TN) and false negative (FN) results for the standard diagnostic test, if available.

The extracted original data were regrouped according to the research purpose. Then, we performed statistical analyses in STATA 14.0 software to obtain the pooled SEN, SPE, positive likelihood ratio (PLR), negative likelihood ratio (NLR), diagnostic odds ratio (DOR) and their corresponding 95% confidence intervals (CIs). We also plotted the summary receiver operating characteristics (sROC) curve to obtain the value of the area under the curve (AUC) and the corresponding 95% CI.

An *I*^2^ value greater than 50% was suggestive of substantial heterogeneity, and then subgroup analysis was performed to identify the source of heterogeneity based on professional knowledge. The existence of a threshold effect was detected by Meta-DiSc software. Publication bias was assessed using Deeks' funnel plots. A sensitivity analysis was used to confirm the stability of the results. A *P-*value <0.05 was considered statistically significant.

## Results

### Characteristics and quality of the included studies

**Fig 1:**
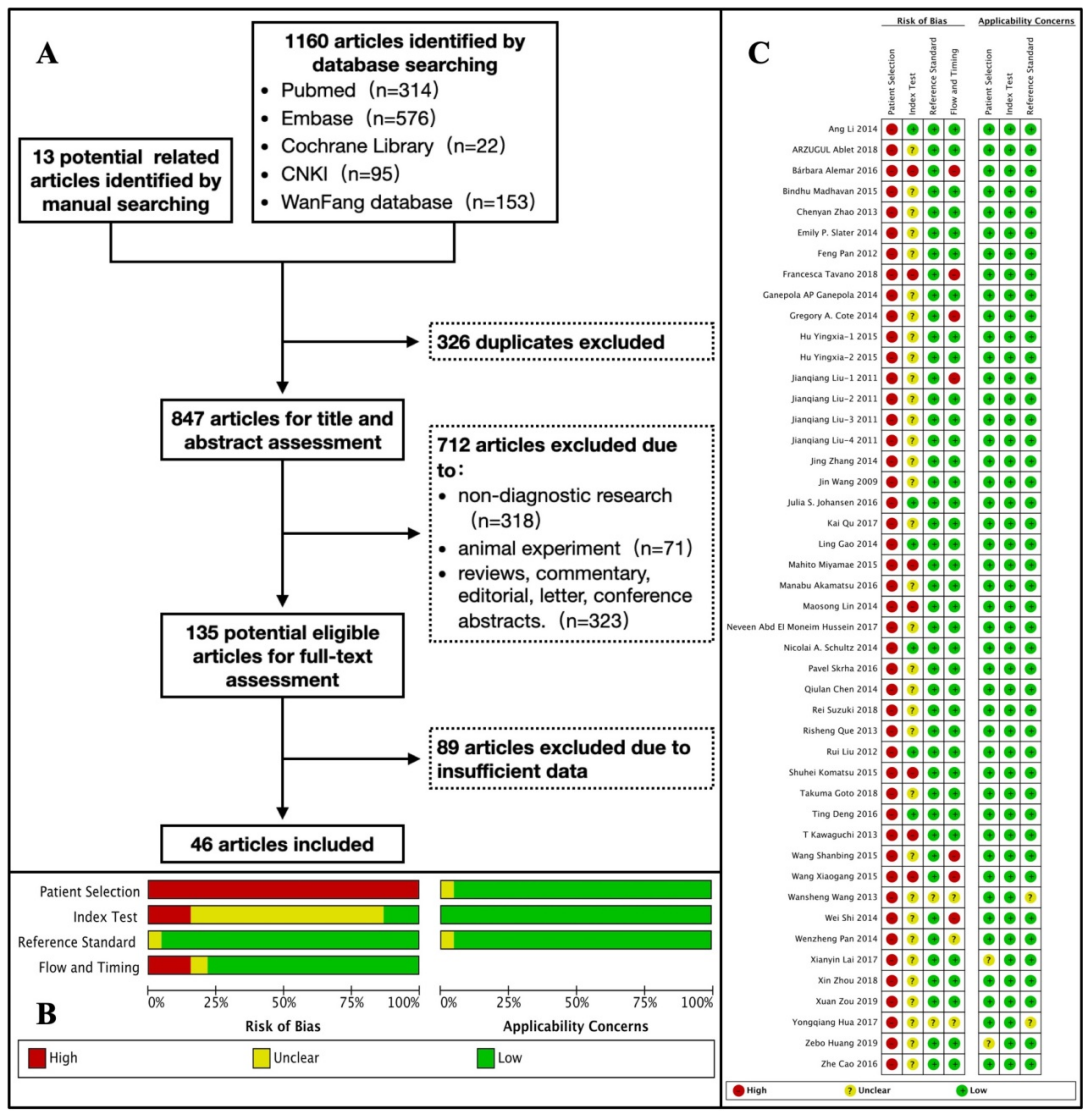
Study selection and quality assessment. (A) Flow diagram of the literature search and study selection process. (B) The review authors' judgment about each domain of risk of bias and applicability concerns presented as percentages across the included studies. (C) The review authors' judgment about each domain of risk of bias and applicability concerns for each included study.

After duplicate removal, title and abstract assessment, and full-text evaluation, we finally included 46 studies involving 4,326 PC patients and 4,277 non-PC controls. The characteristics of the included studies are listed in **Table 1**. Among these original studies, 34 studies were conducted in Asia^13^-^46^, 6 in Europe^47^-^52^, 4 in North America^53^-^56^, 1 in Africa^57^, and 1 in South America^58^. The publication years were 2019 (n=2), 2018 (n=5), 2017 (n=4), 2016 (n=6), 2015 (n=7), 2014 (n=11), 2013 (n=4), 2012 (n=2), 2011 (n=4), and 2009 (n=1). The flow diagram of the literature search and study selection is detailed in **Figure 1 (A)**.

We found that there was a high risk of bias in the domain of "Patient Selection" after the quality assessment using the QUADAS-2 tool. According to the statement of the QUADAS-2 group, an ideal diagnostic study should enroll a proportion of suspected patients ("difficult-to-diagnose patients") to reduce the risk of bias^12^. However, all our included studies included patients with a definitive diagnosis, which resulted in a high risk of bias in this domain. In addition, there was a large proportion of studies with an unclear risk of bias in the domain of the "Index Test" because the researchers of these included studies did not describe how they determined the threshold. The risk of bias was low in the domains of "Reference Test" and "Flow and Timing". All domains exhibited low concerns regarding their applicability. The results of the quality assessment are shown in **Figure 1 (B-C)**.

### Diagnostic performance of circulating miRNAs

**Fig 2:**
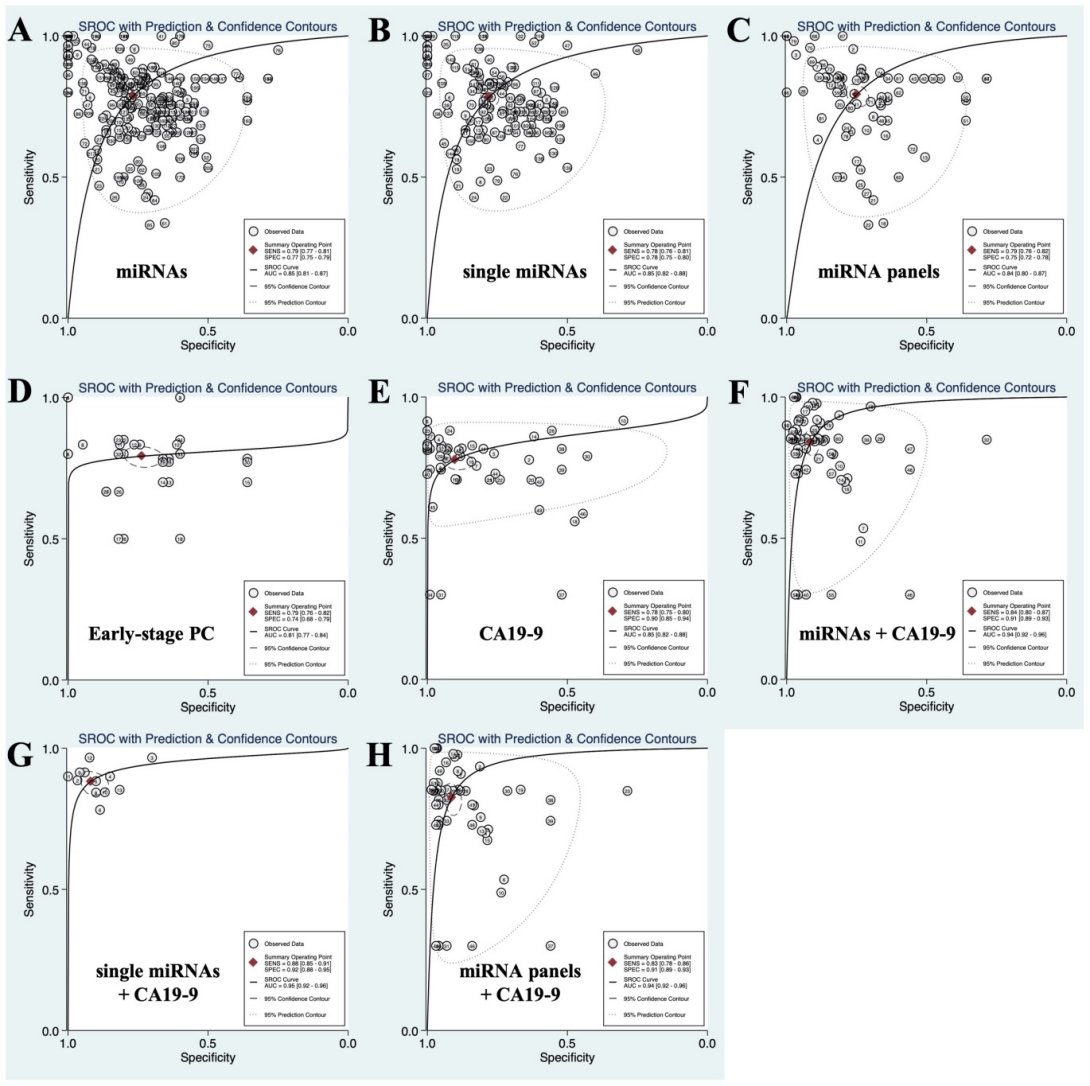
SROC curves describing the diagnostic performance of circulating miRNAs, CA19-9 and the combination of miRNAs and CA19-9 in discriminating PC from non-PC controls. (A) Circulating miRNAs; (B) circulating single miRNAs; (C) circulating miRNA panels; (D) circulating miRNAs for the diagnosis of early-stage PC; (E) CA19-9; (F) the combination of circulating miRNAs and CA19-9; (G) the combination of circulating single miRNAs and CA19-9; (H) the combination of circulating miRNA panels and CA19-9.

Circulating single miRNAs, which means that only one kind of miRNA was used for diagnosis, distinguished PC patients from non-PC controls with a SEN of 0.78 (0.76-0.81) and a SPE of 0.78 (0.75-0.80), and the PLR, NLR, DOR and AUC were 3.55 (3.13-4.02), 0.28 (0.25-0.31), 12.78 (10.19-16.03) and 0.85 (0.82-0.88), respectively. The circulating miRNA panel, which means multiple miRNAs were applied for diagnosis, discriminated PC patients from non-PC controls with a SEN of 0.79 (0.76-0.82), a SPE of 0.75 (0.72-0.78), a PLR of 3.16 (2.74-3.65), a NLR of 0.28 (0.23-0.33), a DOR of 11.40 (8.55-15.20), and an AUC of 0.84 (0.80-0.87). There was no significant difference in the diagnostic efficacy between single miRNAs and miRNA panels. Overall, the SEN, SPE, PLR, NLR, DOR and AUC of circulating miRNAs (including single miRNAs and miRNA panels) in differentiating patients with PC from non-PC controls were 0.79 (0.77-0.81), 0.77 (0.75-0.79), 3.38 (3.08-3.72), 0.28 (0.25-0.31), 12.22 (10.23-14.60) and 0.85 (0.81-0.87), respectively. The results are shown in **Table 2** and **Figure 2 (A-C).**

In addition, we also summarized the SEN, SPE, PLR, NLR, DOR and AUC of miRNAs in distinguishing PC patients from healthy controls (HC) or patients with chronic pancreatitis (CP). The data are listed in **Table 2**. In general, the diagnostic accuracy of miRNAs for discriminating PC from HC was higher than that for discriminating PC from CP.

A total of 58 different single miRNAs and 23 miRNA panels were involved in the 46 included studies. For the single miRNAs and miRNA panels being studied in one data set, we extracted the diagnostic SEN, SPE, PLR, NLR and DOR from the original literature. For those being studied in more than 2 data sets, we performed a meta-analysis and obtained pooled diagnostic SEN, SPE, PLR, NLR and DOR values. The results are listed in **Table S1** and **Table S2**. Among the single miRNAs, miR-122, 212, 22-3p, 483-3p, 642b-3p and 885-5p yielded a high SEN of more than 90%, and the SPE values of miR-25, 223, 17-5p, 223-3p, 30c and 409-3p were greater than 90%. The SEN and SPE of miR-451, miR-106b, miR-10b, miR-181a, miR-196b, miR-20a and let-7a were all greater than 90%. For miRNA panels, the SEN of the combination of let-7b-5p, miR-192-5p, 19a-3p, 19b-3p, 223-3p and 25-3p exceeded 90%, while the SPE of the combination of miR-1246, 4464, 3976 and 4306 was over 90%. The combination of miR-196a and 196b and the combination of miR-451 and 409-3p, as well as the combination of 885-5p, 22-3p and 642b-3p, all exhibited high diagnostic accuracy, with SEN and SPE values greater than 90%.

### Circulating miRNAs for the diagnosis of early-stage PC

Early-stage PC was defined as stage 0-IIa based on the TNM system^18^, ^49^, ^51^, ^55^. For this group of patients, the SEN, SPE, PLR, NLR, DOR and AUC of circulating miRNAs were 0.79 (0.76-0.82), 0.74 (0.68-0.79), 2.60 (2.19-3.10), 0.35 (0.30-0.41), 8.14 (5.85-11.33) and 0.81 (0.77-0.84), respectively. MiR-196b and the combination of miR-196a and 196b exhibited high diagnostic accuracy with SEN and SPE values greater than 90%. The results are listed in **Figure 2 (D)** and **Table 3**.

### Diagnostic performance of conventional biomarkers

In addition to circulating miRNAs, some researchers also evaluated the diagnostic efficacy of conventional biomarkers, such as CA19-9, CEA, and CA242. Among these conventional biomarkers, CA19-9 was the most frequently studied^59^, ^60^. The SEN, SPE, PLR, NLR, DOR and AUC of CA19-9 for discriminating PC patients from non-PC controls were 0.78 (0.75-0.80), 0.90 (0.85-0.94), 7.90 (5.14-12.13), 0.25 (0.22-0.28), 31.89 (18.96-53.62), and 0.85 (0.82-0.88), respectively. The SEN of CEA and CA242 was similar to that of CA19-9, but the SPE was significantly lower than that of CA19-9. CEA distinguished PC patients from non-PC controls with a SEN and a SPE of 0.79 (0.39-0.96) and 0.32 (0.08-0.72), respectively. The PLR, NLR, DOR and AUC of CEA were 1.17 (0.82-1.65), 0.65 (0.26-1.60), 1.80 (0.55-5.88) and 0.59 (0.54-0.63), respectively. The SEN, SPE, PLR, NLR, DOR and AUC of CA242 were 0.79 (0.52-0.93), 0.46 (0.21-0.74), 1.47 (0.95-2.27), 0.45 (0.21-0.97), 3.25 (1.14-9.32) and 0.68 (0.63-0.71), respectively. The results are listed in **Figure 2 (E)** and **Table 2**.

### Diagnostic performance of circulating miRNAs combined with CA19-9

The combination of circulating miRNAs and CA19-9 for the diagnosis of PC exhibited a significantly higher diagnostic accuracy than that of circulating miRNAs or CA19-9 alone. The SEN, SPE, PLR, NLR, DOR, and AUC of miRNAs combined with CA19-9 for differentiating PC patients from non-PC controls were 0.84 (0.80-0.87), 0.84 (0.80-0.87), 9.77 (7.65-12.47), 0.17 (0.14-0.22), 56.01 (37.70-83.20) and 0.94 (0.92-0.96), respectively. The results are listed in **Figure 2 (F-H)** and **Table 2**.

The combination of miR-196, miR-200 and CA19-9 exhibited a high SEN of more than 90%. There were 5 combinations of circulating miRNAs and CA19-9 with diagnostic specificity (SPE) values exceeding 90%: the combination of miR-1290 and CA19-9; the combination of miR-16 and CA19-9; the combination of miR-16, 196a and CA19-9; the combination of miR-145, 150, 223, 636 and CA19-9; and the combination of miR-26b, 34a, 122, 126, 145, 150, 223, 505, 636, 885-5p and CA19-9. There were 4 combinations with SEN and SPE values exceeding 90%, which were the combination of miR-210 and CA19-9; the combination of miR-25 and CA19-9; the combination of miR-196a, 210 and CA19-9; and the combination of miR-181a, 181b, 210 and CA19-9. The results are listed in **Table S3**.

### Subgroup analysis and threshold effect analysis

Since significant heterogeneity was identified in our meta-analysis (*I*^2^>50%), a random-effects model was applied for the pooled analysis. Moreover, subgroup analyses of five potential sources of heterogeneity, namely, region, conference test, miRNA profiling, non-PC control population and specimen, were conducted to identify the source of heterogeneity. However, the results suggested that the *I*^2^ value of most subgroups was still greater than 50%, indicating that these factors were not associated with the heterogeneity. The results are listed in **Table S4**.

The value of the Spearman correlation coefficient was -0.276 (*P*=0.000) in the threshold effect analysis, suggesting the existence of a threshold effect, which might be the main source of heterogeneity in the present meta-analysis.

### Sensitivity analysis and publication bias

A sensitivity analysis was performed to validate the reliability of our results. The removal of any of the original studies did not have a significant impact on the results and corresponding 95% CI, suggesting that the results were stable. Deeks' funnel plots provided no evidence of publication bias (*P*>0.05).

## Discussion

Although the incidence of PC is not high compared with that of other cancers, it is one of the most lethal cancers because of its high invasiveness and rapid progression^61^. It is difficult to diagnose early-stage PC due to the lack of specific clinical manifestations in patients and the absence of auxiliary examination modalities with high sensitivity and specificity. Approximately 50-60% of patients present with distant metastases at the time of diagnosis with PC^62^, which leads to a relatively low five-year survival rate of less than 3%. These data suggest that the prognosis of pancreatic cancer is closely related to the clinical stage at diagnosis^63^. CA19-9 is a tumor antigen that was first discovered in 1979 and has been serving as a PC biomarker for decades^61^. However, a meta-analysis of 19 studies showed insufficient diagnostic accuracy of CA19-9, with pooled SEN and SPE values of 0.78 (0.75-0.81) and 0.73 (0.69-0.76), respectively^64^. Moreover, CA19-9 also exhibited FP results for some non-PC cancers (gastric cancer, ovarian cancer, etc.) and even some benign disorders^65^. In the clinical setting, liquid biopsy has been very popular in recent years because it may complement conventional diagnostic methods. The rationale for liquid biopsy is that tumors can release various forms of substances into body fluids, providing us with an opportunity to detect tumors^66^. Circulating miRNA is one of the biomarkers used in liquid biopsies, and many diagnostic studies on circulating miRNAs are published each year.

In the present meta-analysis, we found that the SEN, SPE and AUC of circulating single miRNAs for discriminating PC patients from non-PC controls were 0.78 (0.76-0.81), 0.78 (0.75-0.80) and 0.85 (0.82-0.88), respectively. The diagnostic performance of the miRNA panels was not significantly improved compared with the performance of single miRNAs. The SEN, SPE and AUC were 0.79 (0.76-0.82), 0.75 (0.72-0.78) and 0.84 (0.80-0.87), respectively.

Overall, the pooled SEN, SPE and AUC of circulating miRNAs (including single miRNAs and miRNA panels) were 0.79 (0.77-0.81), 0.77 (0.75-0.79) and 0.85 (0.81-0.87), respectively. In addition, we also summarized the data for CA19-9 in the included studies and found that the SEN, SPE and AUC of CA19-9 for distinguishing between PC and non-PC were 0.78 (0.75-0.80), 0.90 (0.85-0.94) and 0.85 (0.82-0.88), respectively. The AUC is an indicator that comprehensively reflects the diagnostic efficacy of a biomarker. An AUC of 0.8-0.9 is generally considered to indicate that the diagnostic efficacy is acceptable. An AUC above 0.9 represents a high diagnostic efficacy^67^. The AUCs of both circulating miRNAs and CA19-9 were above 0.8, suggesting that their diagnostic efficacy was acceptable. A promising finding was that the combination of miRNAs and CA19-9 greatly improved the diagnostic accuracy. The pooled SEN, SPE and AUC of the combination were 0.84 (0.80-0.87), 0.91 (0.89-0.93) and 0.94 (0.92-0.96), respectively. Therefore, we concluded that the combination of circulating miRNAs and CA19-9 may be a novel and better strategy for the diagnosis of PC. In addition to the pooled analysis, we also summarized the diagnostic accuracy of all the single miRNAs, miRNA panels and the combinations of miRNAs and CA19-9 involved in the included studies. Some miRNAs and combinations exhibited excellent diagnostic performance. For these miRNAs or combinations, their diagnostic efficacy should be further verified, and their association with the development, progression and prognosis of PC may also be valuable future research topics. Although circulating miRNAs hold promise for the accurate diagnosis of PC and many other tumors, it is not a widely accessible technique in the clinic. Two challenges need to be overcome before large-scale clinical application. (1) Technical challenge: Circulating miRNAs are more difficult to isolate and purify than intracellular miRNAs^68^. In addition, the quantitative methods of circulating miRNAs include reverse transcription-polymerase chain reaction (RT-PCR), microarray and next-generation sequencing (NGS)^69^. Therefore, the technical protocols need to be optimized and standardized. (2) Mechanistic challenge: The functions and regulatory networks of circulating miRNAs in PC remain unclear, and more investigations are needed before clinical application^70^, ^71^.

The early diagnosis of PC has been a problem for a long time. As we mentioned above, the prognosis of patients with PC is related to the stage at diagnosis. The earlier the stage, the higher the 5-year survival rate^4^. In addition, patients with PC who were incidentally diagnosed during imaging examination for unrelated diseases have a longer median survival time than those who are symptomatic^72^. Therefore, it is of great clinical significance to explore the methods of early detection of PC since it is the key issue for improving the prognosis of this aggressive disease. Of the 46 included studies, 4 original studies^18^, ^49^, ^51^, ^55^ proposed the concept of “Early stage PC” and defined it as stage 0-IIa in the TNM staging system. The diagnostic efficacy of relevant circulating miRNAs in this subgroup of PC patients was evaluated. The results showed that circulating miRNAs also exhibited satisfactory diagnostic efficacy in early-stage PC patients, which were defined as PC patients at stage 0-IIa based on the TNM system. The AUC was 0.81 (0.77-0.84), and the SEN and SPE were 0.79 (0.76-0.82) and 0.74 (0.68-0.79), respectively. MiR-196b and the combination of miR-196a and 196b exhibited high diagnostic accuracy, with SEN and SPE values greater than 90%.

Heterogeneity, which is common in diagnostic meta-analyses, is the result of variations among the different included studies^73^. These variations mainly include differences in the study population, study design, interventions and interpretations of results. In general, heterogeneity is derived from the threshold effect and non-threshold effect. Since heterogeneity existed in the present meta-analysis, we first performed a threshold effect analysis, in which the Spearman correlation coefficient was -0.276 (*P*=0.000), indicating the existence of a threshold effect. In addition, we further explored heterogeneous sources of non-threshold effects through subgroup analyses. Based on the available data, we explored the region, conference test, miRNA profiling, non-PC control population and specimen. Unfortunately, the results of the subgroup analysis negated the hypothesis that heterogeneity was caused by these five factors. In summary, we believe that the heterogeneity may be derived from the following aspects: (1) Threshold effect: different circulating miRNAs were involved in the included studies; more importantly, the diagnostic cut-off values also varied, leading to some heterogeneity. (2) Variation in normalization controls: currently, there is no consensus on the selection of the normalization controls when performing the PCR quantification of miRNAs. (3) Location: most of the included studies were conducted in Asia, which may also introduce bias.

The advantages of the present meta-analysis are as follows: (1) we conducted the literature search, study selection and quality assessment in strict accordance with the PRISMA guidelines and ultimately included a total of 46 high-quality studies, and the results were representative; (2) we scientifically grouped the original data according to clinical applicability, making the results more instructive for clinical practice; and (3) we generated a detailed summary in addition to the pooled analysis. The diagnostic accuracy of 58 single miRNAs, 23 miRNA panels and 18 combinations of miRNAs and CA19-9 was summarized, providing evidence-based support for further clinical applications and basic research. However, some limitations also existed in the present meta-analysis: (1) heterogeneity was found in our study, which may affect the reliability of the results to some extent, and (2) not all the included studies avoided using a case-control study design, which is a classic but suboptimal diagnostic study model. According to the statement made by the QUADAS-2 group, a high-accuracy diagnostic study should also enroll some “difficult-to-diagnose” patients; otherwise, the diagnostic performance may be overestimated^12^. Researchers should avoid this issue in subsequent diagnostic studies.

## Conclusion

The results of the present meta-analysis showed that circulating miRNAs yielded a high diagnostic accuracy for PC. More importantly, they also exhibited a satisfactory diagnostic performance for early-stage PC, meeting the urgent need for an ideal biomarker for early-stage PC in clinical settings. The combination of circulating miRNAs and the traditional marker CA19-9 can further improve the diagnostic efficacy, which may be a novel strategy for PC diagnosis. However, the diagnostic efficacy still needs further validation by more high-quality and large-scale diagnostic research.

## Search strategy

(((((((((circulating[Title/Abstract]) OR circulatory[Title/Abstract]) OR serum[Title/Abstract]) OR plasma[Title/Abstract]) OR blood[Title/Abstract]))) AND ((((((((((((Pancreatic Neoplasms[MeSH Terms]) OR Carcinoma, Pancreatic Ductal[MeSH Terms]) OR Pancreatic Intraductal Neoplasms[MeSH Terms]) OR Pancreatic Neoplasms[Title/Abstract]) OR Carcinoma, Pancreatic Ductal[Title/Abstract]) OR Pancreatic Intraductal Neoplasms[Title/Abstract]) OR pancreatic cancer[Title/Abstract]) OR cancer of pancreas[Title/Abstract]) OR pancreatic carcinoma[Title/Abstract]) OR carcinoma of pancreas[Title/Abstract]) OR pancreatic ductal adenocarcinoma[Title/Abstract]) OR PDAC[Title/Abstract])) AND ((((((microRNA[Title/Abstract]) OR microRNAs[Title/Abstract]) OR miRNA[Title/Abstract]) OR miRNAs[Title/Abstract]) OR miR[Title/Abstract]) OR panel[Title/Abstract])) AND (((((((((((((diagnostic[Title/Abstract]) OR diagnosis[Title/Abstract]) OR screen[Title/Abstract]) OR monitor[Title/Abstract]) OR detect[Title/Abstract]) OR predict[Title/Abstract]) OR predictor[Title/Abstract]) OR prediction[Title/Abstract]) OR specificity[Title/Abstract]) OR sensitivity[Title/Abstract]) OR marker[Title/Abstract]) OR AUC[Title/Abstract]) OR detection[Title/Abstract])

## Supplementary Material

Supplementary tables.

## Figures and Tables

**Table 1:** Characteristics of the included studies

Author	Year	Region	Specimen	Conference test	Markers and expression level in PC patients	Normalization controls	PC patients		Non-PC controls	
							No.	Population	No.	Population
Xuan Zou	2019	China	serum	Histopathology	let-7b-5p ↑, miR-192-5p ↑, 19a-3p ↑, 19b-3p ↑, 223-3p ↑, 25-3p ↑	cel-miR-34	159	PC	137	HC
Zebo Huang	2019	China	serum	Histopathology	miR-16 ↑	cel-miR-39	155	PC	137	HC
Takuma Goto	2018	Japan	serum	Imaging	miR-191 ↑, 21 ↑, 451a ↑, CEA ↑, CA19-9 ↑	unclear	32	PC	22	GBP (4), Chronic gastritis (3), Gallbladder stone (2), ADM (2), Liver cyst (1), IBS (1), Accessory spleen (1), Only symptom (7)
Francesca Tavano	2018	Italy	plasma	Histopathology or Imaging	miR-1290 ↑, CA19-9 ↑	unclear	167	PC	267	HC
Rei Suzuki	2018	Japan	serum	Histopathology	miR-let-7d ↓, CEA ↑, CA19-9 ↑	unclear	45	PC	42	CP (18), Biliary stone (20), others (4)
Jin Wang	2018	USA	plasma	Histopathology	miR-21 ↑, 210 ↑, 155 ↑, 196a ↑	miR-16	49	PC	36	HC
Xin Zhou	2018	China	plasma	Histopathology	miR-122-5p ↑, 125b-5p ↑, 192-5p ↑, 193b-3p ↑, 221-3p ↑, 27b-3p ↑	miR-103a	216	PC	220	HC
Arzugul Ablet	2018	China	plasma	Histopathology	miR-21 ↑, 155 ↑	U6	42	PC	84	CP (42), HC (42)
Xianyin Lai	2017	China	plasma	Histopathology	miR-10b ↑, 20a ↑, 21 ↑, 30c ↑, 106b ↑, 181a ↑, let-7a ↓, 122 ↑	miR-425-5p	29	PC	6	HC
Kai Qu	2017	China	serum	Histopathology	miR-21-5p ↑	cel-miR-39	56	PC	15	HC
Yongqiang Hua	2017	China	serum	Unclear	miR-373 ↓	U6	103	PC	50	HC
Neveen Abd EI Moneim Hussein	2017	Egypt	plasma	Histopathology	miR-22-3p ↑, 643b-3p ↑, 885-5p ↑, CA19-9 ↑	miR-3196	35	PC	15	HC
Ting Deng	2016	China	serum	Histopathology	miR-25 ↑	unclear	303	PC	760	HC (600), CP (40), gastric cancer (20), breast cancer (20), lung cancer (20), liver cancer (20), esophageal cancer (20), colorectal cancer (20)
Bárbara Alemar	2016	Brazil	serum	Histopathology	miR-21 ↑, 34a ↑	cel-miR-39	24	PC	9	HC
Zhe Cao	2016	China	plasma	Histopathology	miR-486-5p ↓, 126-3p ↓, 106-3p ↓, 938 ↓, 26b-3p ↓, 1285 ↓, CA19-9 ↑	U6	185	PC	158	CP (73), OPN (85)
Pavel Skrha	2016	Czech Republic	serum	Histopathology	miR-196 ↑, 200 ↑, CA19-9 ↑	miR-191, 454	77	PC	64	HC
Manabu Akamatsu	2016	Japan	serum	Histopathology	miR-7 ↑, 34a ↑, 181d ↑, 193b ↑	cel-miR-39	69	PC	15	AIP
Julia S. Johansen	2016	Denmark	serum	Histopathology	miR-16 ↑, 18a ↓, 24 ↓, 25 ↓, 27a ↓, 30a-5p ↓, 323-3p ↓, 20a ↑, 29c ↓, 191 ↓, 345 ↓, 483-5p ↑, CA19-9 ↑	unclear	417	PC	340	PAC (33), CP (59), HC (248)
Bindhu Madhavan	2015	Germany	serum	Histopathology	miR-1246 ↑, 4644 ↑, 3976 ↑, 4306 ↑	U43, U6, 18S and 5S rRNA	87	PC	51	CP (17), BPT (14), HC (20)
Shuhei Komatsu	2015	Japan	plasma	Histopathology	miR-223 ↑	cel-miR-39	71	PC	67	HC
Mahito Miyamae	2015	Japan	plasma	Histopathology	miR-744 ↑	cel-miR-39	94	PC	68	HC
Hu Yingxia	2015	China	plasma	Histopathology	miR-196a ↑, 210 ↑, CA19-9 ↑	U6	60	PC	30	CP (20), HC (10)
Hu Yingxia	2015	China	plasma	Histopathology	miR-210 ↑, CA19-9 ↑, CA242 ↑, CEA ↑	U6	60	PC	30	CP (20), HC (10)
Wang Xiaogang	2015	China	serum	Histopathology or Imaging	miR-155 ↑, CA19-9 ↑	cel-miR-39	110	PC	70	CP
Wang Shanbing	2015	China	plasma	Histopathology or Imaging	miR-21 ↑, 483-3p ↑, 155 ↑, CA19-9 ↑	miR-16	43	PC	21	HC
Ling Gao	2014	China	plasma	Histopathology	CA19-9 ↑, miR-16 ↑	cel-miR-39	70	PC	120	HC (50), CP (70)
Gregory A. Cote	2014	USA	plasma	Histopathology	miR-10b ↑, 30c ↑, 106b ↑, 155 ↑, 212 ↑	miR-425-5p	40	PC	54	CP (30), BBD (24)
Maosong Lin	2014	China	serum	Histopathology	miR-492 ↓, 663a ↓	cel-miR-39	49	PC	27	HC
Qiulan Chen	2014	China	plasma	Histopathology	miR-182 ↑, CA19-9 ↑	U6	109	PC	38	CP
Ang Li	2014	USA	serum	Histopathology	miR-1290 ↑, 628-3p ↑, 550 ↑, 1825 ↑, 24 ↑, 134 ↑, 146a ↑, 200c ↑, 378 ↑, 484 ↑, 625 ↑, 22 ↑, 210 ↑, 744 ↑, CA19-9 ↑	miR-16	41	PC	72	HC (19), CP (35), pNET (18)
Nicolai A. Schultz	2014	Denmark	serum	Histopathology	miR-145 ↑, 150 ↓, 223 ↑, 636 ↓, 26b ↑, 34a ↑, 122 ↑, 126 ↑, 145 ↑, 150 ↑, 223 ↑, 505 ↑, 636 ↑, 885-5p ↑, CA19-9 ↑	ath-miR-159a	409	PC	347	HC (322), CP (25)
Emily P. Slater	2014	Germany	serum	Histopathology	miR-196a ↑, 196b ↑	miR-24	24	PC	20	CP (10), HC (10)
Ganepola AP Ganepola	2014	USA	plasma	Histopathology	miR-885-5p ↑, 22-3p ↑, 642b-3p ↑, CA19-9 ↑	miR-3196	11	PC	22	HC
Jing Zhang	2014	China	serum	Histopathology	miR-192 ↑, 194 ↑	U6	70	PC	40	HC
Wenzheng Pan	2014	China	plasma	Histopathology	miR-210 ↑, 25 ↑, CA19-9 ↑	cel-miR-39	30	PC	26	HC
Wei Shi	2014	China	plasma	Histopathology or Imaging	miR-155 ↑, 196a ↑, CA19-9 ↑, CA242 ↑, CEA ↑	U6	60	PC	30	CP (20), HC (10)
Risheng Que	2013	China	serum	Histopathology	miR-17-5p ↑, 21 ↑	U6	22	PC	27	AC (6), BPN (7), CP (6), HC (8)
T Kawaguchi	2013	Japan	plasma	Histopathology	miR-221 ↑	U6	47	PC	9	BPN
Wansheng Wang	2013	China	serum	Unclear	miR-27a-3p ↑, CA19-9 ↑	U6	129	PC	163	BPD (103), HC (60)
Chenyan Zhao	2013	China	serum	Histopathology	miR-192 ↑	U6	80	PC	40	HC
Rui Liu	2012	China	serum	Histopathology	miR-20a ↑, 21 ↑, 24 ↑, 25 ↑, 99a ↑, 185 ↑, 191 ↑, CA19-9 ↑, CEA ↑	serum volume	123	PC	61	HC (52), CP (9)
Feng Pan	2012	China	plasma	Histopathology	miR-451 ↑, 409-3p ↑	cel-miR-39	24	PC	24	HC
Jianqiang Liu	2011	China	plasma	Histopathology or Imaging	miR-16 ↑, 196a ↑, CA19-9 ↑	cel-miR-39	138	PC	175	HC (68), CP (107)
Jianqiang Liu	2011	China	plasma	Histopathology	miR-181a ↑, 181b ↑, 210 ↑, CA19-9 ↑	cel-miR-39	55	PC	96	HC (39), CP (57)
Jianqiang Liu	2011	China	plasma	Histopathology	miR-21 ↑	cel-miR-39	45	PC	75	HC (30), CP (45)
Jianqiang Liu	2011	China	plasma	Histopathology	miR-155 ↑	cel-miR-39	62	PC	97	HC (36), CP (61)

***Abbreviations***: PC, pancreatic cancer; HC, healthy control; GBP, gallbladder cholesterol polyp; ADM, adenomyomatosis; IBS, irritable bowel syndrome; CP, chronic pancreatitis; OPN, other pancreatic neoplasms; AIP, autoimmune pancreatitis; PAC, periampullary cancers; BBD, benign biliary disorders; pNET, pancreatic neuroendocrine tumor; AC, ampullary carcinoma; BPN, benign pancreatic neoplasms; BPD, benign pancreatic disease

**Table 2:** The results of meta-analysis

	SEN (95% CI)	SPE (95% CI)	PLR (95% CI)	NLR (95% CI)	DOR (95% CI)	AUC (95% CI)	Number of data sets	Number of PC patients	Number of controls
**1 miRNAs**									
PC vs non-PC	0.79 (0.77-0.81)	0.77 (0.75-0.79)	3.38 (3.08-3.72)	0.28 (0.25-0.31)	12.22 (10.23-14.60)	0.85 (0.81-0.87)	228	13554	14474
PC vs CP	0.77 (0.74-0.80)	0.67 (0.62-0.71)	2.32 (2.01-2.69)	0.35 (0.30-0.40)	6.72 (5.10-8.86)	0.79 (0.75-0.82)	48	2554	1435
PC vs HC	0.83 (0.80-0.85)	0.81 (0.78-0.83)	4.29 (3.67-5.02)	0.22 (0.18-0.26)	19.94 (14.73-26.98)	0.88 (0.85-0.91)	102	5828	5983
**1.1 single miRNAs**									
PC vs non-PC	0.78 (0.76-0.81)	0.78 (0.75-0.80)	3.55 (3.13-4.02)	0.28 (0.25-0.31)	12.78 (10.19-16.03)	0.85 (0.82-0.88)	148	7107	6426
PC vs CP	0.73 (0.68-0.78)	0.68 (0.63-0.73)	2.28 (1.94-2.69)	0.39 (0.32-0.48)	5.80 (4.18-8.03)	0.76 (0.82-0.80)	26	1081	824
PC vs HC	0.81 (0.77-0.85)	0.81 (0.77-0.84)	4.21 (3.46-5.12)	0.23 (0.19-0.29)	17.98 (12.38-26.10)	0.88 (0.85-0.90)	72	3756	2686
**1.2 miRNA panel**									
PC vs non-PC	0.79 (0.76-0.82)	0.75 (0.72-0.78)	3.16 (2.74-3.65)	0.28 (0.23-0.33)	11.40 (8.55-15.20)	0.84 (0.80-0.87)	80	6447	8048
PC vs CP	0.80 (0.77-0.83)	0.65 (0.56-0.73)	2.30 (1.79-2.95)	0.30 (0.25-0.37)	7.58 (4.91-11.70)	0.82 (0.78-0.85)	22	1473	611
PC vs HC	0.86 (0.83-0.88)	0.81 (0.76-0.85)	4.47 (3.43-5.81)	0.18 (0.14-0.22)	25.43 (16.02-40.37)	0.90 (0.88-0.93)	30	2072	3297
**2 miRNAs combined with CA19-9**									
PC vs non-PC	0.84 (0.80-0.87)	0.91 (0.89-0.93)	9.77 (7.65-12.47)	0.17 (0.14-0.22)	56.01 (37.70-83.20)	0.94 (0.92-0.96)	65	6121	8124
PC vs CP	0.82 (0.76-0.87)	0.82 (0.73-0.89)	4.61 (2.87-7.40)	0.22 (0.15-0.32)	21.12 (9.59-46.51)	0.89 (0.86-0.91)	16	1280	562
PC vs HC	0.86 (0.81-0.91)	0.96 (0.94-0.97)	19.52 (14.92-25.53)	0.14 (0.10-0.20)	136.75 (91.16-205.15)	0.97 (0.96-0.98)	20	1725	3106
**2.1 single miRNAs combined with CA19-9**									
PC vs non-PC	0.88 (0.85-0.91)	0.92 (0.88-0.95)	10.80 (7.12-16.38)	0.13 (0.10-0.17)	84.16 (47.15-150.25)	0.95 (0.92-0.96)	12	965	830
PC vs CP	0.83 (0.79-0.87)	0.88 (0.83-0.92)	7.18 (4.87-10.58)	0.19 (0.14-0.24)	38.29 (22.55-65.00)	0.92 (0.90-0.94)	4	349	198
PC vs HC	0.92 (0.87-0.96)	0.94 (0.87-0.97)	15.30 (6.88-34.01)	0.08 (0.05-0.14)	189.00 (89.48-399.17)	0.97 (0.95-0.98)	5	357	379
**2.2 miRNA panel combined with CA19-9**									
PC vs non-PC	0.83 (0.78-0.86)	0.91 (0.89-0.93)	9.49 (7.14-12.61)	0.19 (0.15-0.25)	49.60 (31.15-78.98)	0.94 (0.92-0.96)	53	5156	7294
PC vs CP	0.81 (0.70-0.88)	0.79 (0.66-0.88)	3.88 (2.12-7.08)	0.24 (0.14-0.42)	16.04 (5.41-47.54)	0.87 (0.84-0.90)	12	931	364
PC vs HC	0.83 (0.75-0.89)	0.96 (0.94-0.97)	20.40 (15.17-27.45)	0.17 (0.12-0.26)	116.62 (71.44-190.38)	0.97 (0.95-0.98)	15	1368	2727
**3 Conventional biomarker (PC vs non-PC)**									
CA19-9	0.78 (0.75-0.80)	0.90 (0.85-0.94)	7.90 (5.14-12.13)	0.25 (0.22-0.28)	31.89 (18.96-53.62)	0.85 (0.82-0.88)	51	3787	4508
CEA	0.79 (0.39-0.96)	0.32 (0.08-0.72)	1.17 (0.82-1.65)	0.65 (0.26-1.60)	1.80 (0.55-5.88)	0.59 (0.54-0.63)	10	500	237
CA242	0.79 (0.52-0.93)	0.46 (0.21-0.74)	1.47 (0.95-2.27)	0.45 (0.21-0.97)	3.25 (1.14-9.32)	0.68 (0.63-0.71)	5	300	90
CA19-9, CEA, CA242	0.77 (0.61-0.88)	0.66 (0.42-0.85)	2.29 (1.15-4.58)	0.35 (0.18-0.67)	6.61 (1.92-22.77)	0.79 (0.75-0.82)	5	300	90

***Abbreviations***: PC, pancreatic cancer; HC, healthy control; CP, chronic pancreatitis; SEN, sensitivity; SPE, specificity; PLR, positive likelihood ratio; NLR, negative likelihood ratio; DOR, diagnostic odds ratio; AUC, area under the curve.

**Table 3:** The diagnostic performance of circulating miRNAs for early-stage PC

MiRNAs	TNM stage	Number of data sets	Number of PC	Number of non-PC	SEN (95% CI)	SPE (95% CI)	PLR (95% CI)	NLR (95% CI)	DOR (95% CI)
miR-196a	0	2	10	20	1.00 (0.69-1.00)	0.60 (0.36-0.81)	2.24 (1.32-3.81)	0.14 (0.02-0.95)	15.89 (1.73-145.79)
miR-196b	0	2	10	20	0.90 (0.56-1.00)	1.00 (0.83-1.00)	18.26 (2.64-126.12)	0.21 (0.06-0.71)	107.46 (7.99-1444.70)
miR-196a, 196b	0	2	10	20	0.90 (0.56-1.00)	1.00 (0.83-1.00)	18.26 (2.64-126.12)	0.21 (0.06-0.71)	107.46 (7.99-1444.70)
miR-1290	I	5	30	198	0.83 (0.65-0.94)	0.78 (0.71-0.83)	3.45 (2.39-4.99)	0.22 (0.10-0.49)	18.21 (6.31-52.56)
miR-191	I-IIa	1	9	22	0.67	0.84	4.22	0.40	10.67
miR-21	I-IIa	1	9	22	0.67	0.81	3.51	0.41	8.54
miR-451a	I-IIa	1	9	22	0.67	0.86	4.66	0.39	12.00
miR-145, 150, 223, 636	I-IIa	9	420	2082	0.77 (0.73-0.81)	0.64 (0.62-0.66)	1.83 (1.54-2.18)	0.40 (0.33-0.48)	4.65 (3.26-6.64)
miR-26b, 34a, 122, 126, 145, 150, 223, 505, 636, 885-5p	I-IIa	9	420	2082	0.80 (0.76-0.84)	0.80 (0.79-0.82)	3.23 (2.55-4.09)	0.33 (0.23-0.48)	10.20 (6.03-17.26)
Overall	0-IIa	32	927	4488	0.79 (0.76-0.82)	0.74 (0.68-0.79)	2.60 (2.19-3.10)	0.35 (0.30-0.41)	8.14 (5.85-11.33)

***Abbreviations***: PC, pancreatic cancer; SEN, sensitivity; SPE, specificity; PLR, positive likelihood ratio; NLR, negative likelihood ratio; DOR, diagnostic odds ratio.

## Abbreviations

PC: pancreatic cancer
USPSTF: US Preventive Services Task Force
CT: computed tomography
MRI: magnetic resonance imaging
US: ultrasound
CA19-9: carbohydrate antigen 19-9
EUS: endoscopic ultrasonography
EUS-FNA: EUS-guided fine-needle aspiration
CTCs: circulating tumor cells
cfDNAs: cell-free circulating tumor DNAs
miRNAs: microRNAs
lncRNAs: long noncoding RNAs
TEPs: tumor educated platelets
CNKI: China National Knowledge Infrastructure
QUADAS-2: Quality Assessment of Diagnostic Accuracy Studies 2
TP: true positive
TN: true negative
FP: false positive
FN: false negative
SEN: sensitivity
SPE: specificity
PLR: positive likelihood ratio
NLR: negative likelihood ratio
DOR: diagnostic odds ratio
CI: confidence interval
sROC: summary receiver operating characteristics curve
AUC: area under the curve
RT-PCR: reverse transcription-polymerase chain reaction
NGS: next-generation sequencing
