# Supplementary material for: Meta-analysis of the Diagnostic Performance of Circulating MicroRNAs for Pancreatic Cancer
Source: Int J Med Sci. 2021 Jan 1;18(3):660–71. doi: 10.7150/ijms.52706 (PMC7797557; doi:10.7150/ijms.52706)
Supplement: Supplementary file 1 — Supplementary tables. [file ijmsv18p0660s1.pdf]

Table S1 The summarization of diagnostic accuracy of circulating single miRNAs in discriminating PC from non-PC controls

MiRNAs	Number of data sets	Number of PC	Number of controls	SEN (95% CI)	SPE (95% CI)	PLR (95% CI)	NLR (95% CI)	DOR (95% CI)
106b	2	69	60	0.99 (0.92-1.00)	0.98 (0.91-1.00)	27.95 (7.18-108.81)	0.03 (0.01-0.22)	869.87 (77.31-9787.60)
10b	2	69	60	0.97 (0.90-1.00)	1.00 (0.94-1.00)	36.60 (4.89-274.06)	0.05 (0.02-0.15)	1257.70 (110.18-14357.4)
122	1	29	6	1.00	0.67	3.03	0.00	—
1290	10	455	930	0.83 (0.78-0.88)	0.76 (0.71-0.80)	3.17 (2.61-3.84)	0.23 (0.17-0.31)	14.14 (8.97-22.29)
134	3	123	108	0.71 (0.62-0.79)	0.60 (0.50-0.70)	1.71 (1.32-2.23)	0.49 (0.36-0.67)	3.58 (2.04-6.27)
146a	3	123	108	0.76 (0.68-0.84)	0.75 (0.66-0.83)	3.01 (2.14-4.24)	0.31 (0.22-0.43)	10.19 (5.45-19.05)
155	9	548	405	0.71 (0.67-0.75)	0.81 (0.76-0.84)	2.88 (1.86-4.46)	0.37 (0.25-0.53)	8.49 (4.02-17.94)
16	4	365	377	0.69 (0.64-0.73)	0.75 (0.71-0.80)	2.84 (2.36-3.42)	0.30 (0.14-0.65)	9.41 (4.25-20.83)
17-5p	1	22	27	0.73	0.93	9.82	0.30	33.32
181a	1	29	6	0.97	1.00	—	0.03	—
181d	1	69	15	0.81	0.80	4.05	0.24	17.05
182	1	109	38	0.64	0.83	3.68	0.44	8.48
1825	5	205	198	0.69 (0.63-0.76)	0.65 (0.58-0.71)	1.93 (1.56-2.39)	0.47 (0.38-0.60)	4.19 (2.71-6.49)
191	3	64	66	0.73 (0.61-0.84)	0.83 (0.72-0.91)	4.20 (2.40-7.36)	0.32 (0.21-0.49)	13.70 (5.66-33.15)
192	2	150	80	0.76 (0.68-0.83)	0.55 (0.44-0.66)	1.69 (1.30-2.19)	0.44 (0.31-0.62)	3.87 (2.17-6.90)
192-5p	1	129	101	0.78	0.57	1.80	0.40	4.57
193b	1	69	15	0.79	0.73	2.93	0.29	10.17
194	1	70	40	0.58	0.55	1.29	0.76	1.69
196a	9	217	126	0.67 (0.60-0.73)	0.77 (0.69-0.84)	2.61 (1.77-3.84)	0.33 (0.18-0.61)	11.60 (4.12-32.69)
196b	6	48	60	0.94 (0.83-0.99)	0.92 (0.82-0.97)	5.92 (2.66-13.19)	0.14 (0.07-0.31)	71.67 (18.90-271.77)
19a-3p	1	129	101	0.71	0.79	3.32	0.37	9.07
19b-3p	1	129	101	0.65	0.81	3.48	0.43	8.11
200c	3	123	108	0.66 (0.57-0.74)	0.61 (0.51-0.70)	1.68 (1.28-2.21)	0.55 (0.41-0.74)	3.08 (1.77-5.34)

20a	1	29	6	0.93	1.00	—	0.07	—
21	11	366	315	0.75 (0.71-0.80)	0.77 (0.72-0.82)	3.14 (2.45-4.02)	0.29 (0.20-0.42)	12.05 (6.95-20.89)
21-5p	1	56	15	0.77	0.80	3.85	0.29	13.39
210	6	300	141	0.80 (0.75-0.84)	0.57 (0.49-0.66)	1.62 (1.24-2.12)	0.36 (0.20-0.64)	5.36 (2.64-10.86)
212	1	40	54	0.90	0.83	5.29	0.12	43.94
22	1	41	19	0.71	0.79	3.38	0.37	9.21
22-3p	2	46	37	0.94 (0.82-0.99)	0.87 (0.71-0.96)	6.66 (1.73-25.71)	0.09 (0.01-0.67)	81.40 (3.77-1759.40)
221	1	47	9	0.74	0.78	3.36	0.33	10.09
223	1	71	67	0.62	0.94	10.51	0.40	26.02
223-3p	1	129	101	0.78	0.92	9.32	0.24	39.35
24	3	123	108	0.71 (0.61-0.79)	0.70 (0.61-0.79)	2.29 (1.68-3.12)	0.43 (0.32-0.58)	5.48 (3.08-9.76)
25	2	333	786	0.75 (0.70-0.80)	0.92 (0.90-0.94)	5.63 (1.45-21.80)	0.27 (0.22-0.33)	19.31 (3.64-102.45)
25-3p	1	129	101	0.67	0.80	3.40	0.41	8.22
27a-3p	2	258	163	0.83 (0.77-0.87)	0.79 (0.72-0.85)	3.90 (2.85-5.34)	0.22 (0.17-0.29)	18.15 (10.95-30.09)
30c	2	69	60	0.84 (0.73-0.92)	0.97 (0.89-1.00)	18.19 (5.36-61.69)	0.10 (0.01-1.48)	110.70 (16.64-736.34)
34a	2	93	24	0.84 (0.75-0.91)	0.79 (0.58-0.93)	4.09 (1.87-8.95)	0.20 (0.10-0.39)	21.95 (6.80-70.85)
373	1	103	50	0.81	0.84	5.13	0.23	22.31
378	3	123	108	0.72 (0.63-0.79)	0.69 (0.60-0.78)	2.25 (1.65-3.07)	0.41 (0.30-0.56)	5.56 (3.11-10.00)
409-3p	1	24	24	0.58	0.92	7.02	0.46	15.45
451	1	24	24	0.92	0.92	11.05	0.09	122.06
451a	3	64	66	0.67 (0.54-0.78)	0.85 (0.74-0.93)	4.39 (2.41-8.00)	0.39 (0.27-0.56)	11.47 (4.77-27.57)
483-3p	1	43	21	0.98	0.62	2.56	0.04	69.01
484	3	123	108	0.73 (0.64-0.81)	0.65 (0.55-0.74)	2.08 (1.57-2.75)	0.42 (0.30-0.58)	5.07 (2.85-9.02)
492	1	49	27	0.76	0.70	2.52	0.35	7.19
550	5	205	198	0.60 (0.53-0.67)	0.66 (0.59-0.73)	1.67 (1.33-2.09)	0.64 (0.53-0.78)	2.75 (1.80-4.20)
625	3	123	108	0.68 (0.59-0.76)	0.58 (0.49-0.68)	1.64 (1.27-2.12)	0.54 (0.39-0.74)	3.08 (1.77-5.36)

628-3p	5	205	198	0.73 (0.67-0.79)	0.65 (0.58-0.72)	2.00 (1.61-2.50)	0.41 (0.32-0.53)	4.96 (3.17-7.76)
642b-3p	2	46	37	0.96 (0.85-1.00)	0.73 (0.56-0.86)	6.84 (0.04-1158.40)	0.08 (0.00-2.59)	83.80 (0.21-33509.60)
663a	1	49	27	0.86	0.80	4.26	0.18	23.97
7	1	69	15	0.72	0.73	2.67	0.38	6.95
744	2	135	87	0.62 (0.54-0.70)	0.82 (0.72-0.89)	2.83 (0.63-12.82)	0.47 (0.37-0.60)	5.75 (1.10-29.92)
885-5p	2	46	37	0.96 (0.85-1.00)	0.84 (0.68-0.94)	8.33 (0.23-301.87)	0.08 (0.00-1.56)	120.00 (0.70-20570.70)
let-7a	1	29	6	0.93	1.00	—	0.07	—
let-7b-5p	1	129	101	0.80	0.60	1.99	0.34	5.88
let-7d	1	45	42	0.89	0.68	2.80	0.16	17.18

Abbreviations: PC, pancreatic cancer; SEN, sensitivity; SPE, specificity; PLR, positive likelihood ratio; NLR, negative likelihood ratio; DOR, diagnostic odds ratio.

Table S2 The summarization of diagnostic accuracy of circulating miRNAs panels in discriminating PC from non-PC controls

MiRNA panel	Number of data sets	Number of PC	Number of controls	SEN (95% CI)	SPE (95% CI)	PLR (95% CI)	NLR (95% CI)	DOR (95% CI)
let-7b-5p, miR-192-5p, 19a-3p, 19b-3p, 223-3p, 25-3p	2	159	131	0.95 (0.90-0.98)	0.81 (0.73-0.87)	8.59 (1.17-62.94)	0.06 (0.03-0.12)	113.07 (22.13-577.74)
122-5p, 125b-5p, 192-5p, 193b-3p, 221-3p, 27b-3p	3	216	220	0.83 (0.78-0.88)	0.79 (0.73-0.84)	4.07 (2.44-6.78)	0.20 (0.13-0.33)	21.52 (7.87-58.83)
1246, 4644, 3976, 4306	1	87	51	0.81	0.94	13.50	0.20	66.79
145, 150, 223, 636	16	1287	2557	0.83 (0.80-0.85)	0.64 (0.62-0.66)	1.89 (1.63-2.20)	0.33 (0.27-0.40)	5.78 (4.09-8.18)
155, 196a	2	120	30	0.68 (0.59-0.77)	0.60 (0.41-0.77)	1.64 (0.89-3.03)	0.50 (0.23-1.09)	3.38 (0.84-13.53)
16, 18a, 24, 27a, 30a-5p, 323-3p, 20a, 25, 29c, 191, 345, 483-5p	3	417	340	0.67 (0.62-0.71)	0.80 (0.76-0.84)	2.68 (0.99-7.27)	0.41 (0.14-1.20)	6.66 (0.78-57.10)
16, 196a	3	414	350	0.76 (0.71-0.80)	0.74 (0.69-0.79)	2.75 (2.17-3.50)	0.32 (0.20-0.52)	8.69 (4.71-16.03)
16, 24, 27a, 30a-5p, 323-3p, 20a, 25, 29c, 483-5p	3	417	340	0.61 (0.57-0.66)	0.77 (0.72-0.81)	2.23 (1.14-4.35)	0.55 (0.30-1.01)	4.10 (1.12-14.96)
16, 27a, 25, 29c, 483-5p	3	417	340	0.56 (0.51-0.61)	0.76 (0.71-0.81)	2.20 (1.21-3.99)	0.56 (0.31-1.01)	3.94 (1.28-12.07)
16, 27a, 30a-5p, 323-3p, 20a, 29c, 483-5p	2	219	156	0.47 (0.40-0.54)	0.70 (0.62-0.77)	1.47 (0.65-3.32)	0.78 (0.46-1.31)	1.91 (0.50-7.23)
181a, 181b, 210	3	165	192	0.81 (0.74-0.87)	0.87 (0.81-0.91)	6.07 (2.37-15.54)	0.22 (0.09-0.53)	37.43 (6.35-220.83)
192, 194	1	70	40	0.84	0.75	3.36	0.21	15.75
196a, 196b	6	48	60	0.98 (0.89-1.00)	0.95 (0.86-0.99)	7.51 (3.58-15.77)	0.11 (0.05-0.28)	144.80 (33.27-630.32)
196a, 210	1	60	30	0.88	0.73	3.31	0.16	20.72
20a, 21, 24, 25, 99a, 185, 191	2	123	61	0.88 (0.81-0.93)	0.82 (0.70-0.91)	4.17 (1.94-8.95)	0.16 (0.09-0.26)	28.27 (9.83-81.34)
21, 155	3	126	168	0.71 (0.63-0.79)	0.69 (0.62-0.76)	2.31 (1.79-2.97)	0.42 (0.31-0.56)	5.61 (3.35-9.40)
21, 210, 155, 196a	1	49	36	0.64	0.89	5.82	0.40	14.38
26b, 34a, 122, 126, 145, 150, 223, 505, 636, 885-5p	16	1287	2557	0.83 (0.81-0.85)	0.80 (0.78-0.81)	3.05 (2.37-3.93)	0.27 (0.22-0.34)	12.00 (7.82-18.43)

451, 409-3p	1	24	24	0.96	0.92	11.54	0.05	252.00
486-5p, 126-3p, 106-3p	3	341	158	0.70 (0.65-0.75)	0.65 (0.57-0.73)	2.57 (0.77-8.61)	0.39 (0.14-1.08)	6.70 (0.82-54.81)
486-5p, 126-3p, 106-3p, 938, 26b-3p, 1285	3	341	158	0.74 (0.69-0.79)	0.72 (0.65-0.79)	3.03 (1.49-6.15)	0.31 (0.15-0.63)	10.12 (2.57-39.89)
492, 663a	1	49	27	0.86	0.80	4.29	0.18	23.97
885-5p, 22-3p, 642b-3p	1	11	22	0.91	0.91	10.11	0.10	102.24

Abbreviations: PC, pancreatic cancer; SEN, sensitivity; SPE, specificity; PLR, positive likelihood ratio; NLR, negative likelihood ratio; DOR, diagnostic odds ratio.

Table S3 The summarization of diagnostic accuracy of the combination of circulating miRNAs and CA19-9 in discriminating PC from non-PC controls

MiRNAs combined with CA19-9	Number of data sets	Number of PC patients	Number of controls	SEN (95% CI)	SPE (95% CI)	PLR (95% CI)	NLR (95% CI)	DOR (95% CI)
miR-1290, CA19-9	1	167	267	0.89	0.97	26.06	0.12	220.81
miR-155, CA19-9	1	110	70	0.78	0.89	7.09	0.25	28.69
miR-16, CA19-9	3	210	240	0.89 (0.84-0.93)	0.94 (0.90-0.97)	13.38 (7.25-24.67)	0.12 (0.08-0.19)	117.50 (41.03-336.52)
miR-182, CA19-9	1	109	38	0.85	0.87	6.42	0.18	36.40
miR-210, CA19-9	4	210	86	0.91 (0.87-0.95)	0.90 (0.81-0.95)	6.34 (2.90-13.90)	0.11 (0.07-0.17)	71.72 (29.96-171.69)
miR-25, CA19-9	1	30	26	0.97	0.92	12.56	0.04	351.26
miR-27a-3p, CA19-9	1	129	103	0.85	0.82	4.64	0.18	25.73
miR-16, 196a, CA19-9	3	414	350	0.89 (0.86-0.92)	0.97 (0.94-0.98)	27.10 (15.14-48.49)	0.12 (0.09-0.15)	240.07 (122.04-472.26)
miR-196, 200, CA19-9	1	77	64	0.94	0.82	5.22	0.07	71.37
miR-196a, 210, CA19-9	1	60	30	0.95	0.93	14.18	0.05	264.58
miR-145, 150, 223, 636, CA19-9	16	1287	2557	0.82 (0.79-0.84)	0.93 (0.92-0.94)	6.44 (3.54-11.74)	0.29 (0.20-0.41)	23.95 (11.63-49.33)
miR-16, 18a, 24, 25, 27a, 30a-5p, 323-3p, 20a, 29c, 191, 345, 483-5p, CA19-9	1	198	184	0.71	0.78	3.23	0.37	8.68
miR-16, 18a, 24, 27a, 30a-5p, 323-3p, 20a, 25, 29c, 191, 345, 483-5p, CA19-9	3	417	340	0.83 (0.79-0.87)	0.86 (0.81-0.89)	4.95 (2.25-10.88)	0.17 (0.04-0.62)	31.57 (3.56-279.72)
miR-16, 24, 27a, 30a-5p, 323-3p, 20a, 25, 29c, 483-5p, CA19-9	3	417	340	0.85 (0.81-0.88)	0.86 (0.82-0.89)	5.50 (3.40-8.90)	0.19 (0.09-0.39)	29.06 (8.76-96.421)
miR-16, 27a, 25, 29c, 483-5p, CA19-9	3	417	340	0.82 (0.78-0.85)	0.87 (0.82-0.90)	5.46 (1.66-18.03)	0.09 (0.00-4.30)	62.80 (1.65-2384.20)
miR-16, 27a, 30a-5p, 323-3p, 20a, 29c, 483-5p, CA19-9	3	417	340	0.77 (0.72-0.81)	0.82 (0.78-0.86)	3.69 (1.59-8.54)	0.30 (0.11-0.83)	12.56 (1.96-80.27)
miR-181a, 181b, 210, CA19-9	3	165	192	1.00 (0.98-1.00)	0.97 (0.93-0.99)	25.68 (12.73-51.81)	0.01 (0.00-0.05)	2751.30 (463.65-16326.10)
miR-26b, 34a, 122, 126, 145, 150, 223, 505, 636, 885-5p, CA19-9	16	1287	2557	0.81 (0.79-0.83)	0.96 (0.95-0.97)	11.33 (7.53-17.06)	0.26 (0.19-0.36)	46.20 (26.60-80.23)

Abbreviations: PC, pancreatic cancer; SEN, sensitivity; SPE, specificity; PLR, positive likelihood ratio; NLR, negative likelihood ratio; DOR, diagnostic odds ratio.

Table S4 The results of subgroup analysis

	SEN (95% CI)	SPE (95% CI)	PLR (95% CI)	NLR (95% CI)	DOR (95% CI)	AUC (95% CI)	I^2— OR	Number of data sets	Number of PC patients	Number of controls
**1 Region**										
Asia	0.80 (0.77-0.82)	0.78 (0.75-0.81)	3.68 (3.23-4.19)	0.25 (0.22-0.29)	14.47 (11.41-18.34)	0.86 (0.83-0.89)	76.3%	97	6583	5015
Europe	0.80 (0.75-0.83)	0.76 (0.71-0.80)	3.27 (2.71-3.95)	0.27 (0.22-0.34)	12.09 (8.38-17.45)	0.84 (0.81-0.87)	83.4%	64	4495	7055
Africa	0.99 (0.95-1.00)	0.98 (0.88-1.00)	21.29 (5.51-82.27)	0.02 (0.01-0.08)	1033.90 (140.55-7605.60)	-	0.0%	3	105	45
South America	0.88 (0.75-0.95)	0.78 (0.52-0.94)	3.93 (4.65-9.40)	0.17 (0.08-0.38)	24.80 (5.99-102.73)	-	0.0%	2	48	18
North America	0.73 (0.69-0.76)	0.74 (0.70-0.77)	2.76 (2.34-3.25)	0.37 (0.32-0.43)	7.48 (5.56-10.05)	0.79 (0.76-0.83)	56.2%	63	2323	2341
**2 Specimen**										
Serum	0.77 (0.75-0.79)	0.74 (0.72-0.76)	2.96 (2.69-3.24)	0.31 (0.28-0.34)	9.53 (8.00-11.36)	0.82 (0.79-0.85)	77.8%	153	9192	10930
Plasma	0.82 (0.78-0.86)	0.82 (0.78-0.86)	4.67 (3.72-5.87)	0.21 (0.17-0.27)	21.77 (14.25-33.27)	0.89 (0.86-0.92)	76.3%	76	4362	3544
**3 MiRNA profiling**										
single miRNAs	0.78 (0.76-0.81)	0.78 (0.75-0.80)	3.55 (3.13-4.02)	0.28 (0.25-0.31)	12.78 (10.19-16.03)	0.85 (0.82-0.88)	68.5%	149	7107	6426
miRNAs panel	0.79 (0.76-0.82)	0.75 (0.72-0.78)	3.16 (2.74-3.65)	0.28 (0.23-0.33)	11.40 (8.55-15.20)	0.84 (0.80-0.87)	85.2%	80	6447	8048
**4 Conference test**										
Histopathology	0.79 (0.77-0.81)	0.77 (0.75-0.79)	3.45 (3.11-3.84)	0.27 (0.24-0.30)	12.72 (10.39-15.59)	0.85 (0.82-0.88)	78.7%	199	11588	12896
Imaging	0.74 (0.67-0.80)	0.83 (0.77-0.88)	4.44 (3.21-6.13)	0.31 (0.24-0.40)	14.20 (8.67-23.26)	0.86 (0.83-0.89)	0.0%	9	192	198
Histopathology or Imaging	0.78 (0.70-0.85)	0.69 (0.60-0.77)	2.51 (1.96-3.22)	0.32 (0.23-0.43)	7.94 (5.12-12.32)	0.80 (0.76-0.83)	67.5%	18	1413	1167
Unclear	0.82 (0.78-0.86)	0.80 (0.74-0.85)	4.08 (3.10-5.37)	0.22 (0.18-0.28)	18.96 (12.21-29.46)	-	0.0%	3	361	213
**5 Non-PC control population**										
HC	0.83 (0.80-0.85)	0.81 (0.78-0.83)	4.29 (3.67-5.02)	0.22 (0.18-0.26)	19.94 (14.73-26.98)	0.88 (0.85-0.91)	70.5%	103	5828	5983
CP	0.77 (0.74-0.80)	0.67 (0.62-0.71)	2.32 (2.01-2.69)	0.35 (0.30-0.40)	6.72 (5.10-8.86)	0.79 (0.75-0.82)	56.5%	48	2554	1435
HC+CP	0.74 (0.69-0.78)	0.72 (0.69-0.75)	2.64 (2.31-3.03)	0.36 (0.31-0.43)	7.25 (5.49-9.57)	0.79 (0.75-0.82)	80.2%	37	2853	4553

Other disease	0.75 (0.70-0.80)	0.81 (0.76-0.85)	3.96 (3.01-5.20)	0.30 (0.24-0.38)	13.04 (8.08-21.02)	0.85 (0.82-0.88)	87.1%	41	2319	2503

Abbreviations: PC, pancreatic cancer; HC, healthy control; CP, chronic pancreatitis; SEN, sensitivity; SPE, specificity; PLR, positive likelihood ratio; NLR, negative likelihood ratio; DOR, diagnostic odds ratio; AUC, area under the curve.
